# Oxaliplatin-Induced Pulmonary Toxicity in Gastrointestinal Malignancies: Two Case Reports and Review of the Literature

**DOI:** 10.1155/2015/341064

**Published:** 2015-05-10

**Authors:** Mor Moskovitz, Mira Wollner, Nissim Haim

**Affiliations:** Division of Oncology, Rambam Health Care Campus, 3109601 Haifa, Israel

## Abstract

Oxaliplatin is a common chemotherapy drug, used mainly for colon and gastric cancer. Most common side effects are peripheral sensory neuropathy, hematological toxicity, and allergic reactions. A less common side effect is pulmonary toxicity, characterized mainly by interstitial pneumonitis. The incidence of this side effect is unknown, but the toxicity can be fatal. Twenty-six cases of pulmonary toxicity have been described in the literature, seven in the setting of adjuvant treatment. We describe two fatal cases of pulmonary injury related to oxaliplatin and a review of the literature.

## 1. Introduction

Oxaliplatin is a common chemotherapy drug of the platinum salts class and is used for the treatment of colon cancer and other gastrointestinal malignancies, usually in combination with 5-fluorouracil [1, 2]. The main dose-limiting toxicities of oxaliplatin are peripheral sensory neuropathy and hematological toxicity [3]. A less common side effect is pulmonary toxicity, characterized mainly by interstitial pneumonitis. The incidence of this side effect is unknown, as well as the risk factors, but the pulmonary toxicity can be fatal. Hereby we present two fatal cases of pulmonary injury related to oxaliplatin and a review of the literature.

## 2. Case 1

**Figure 1:**
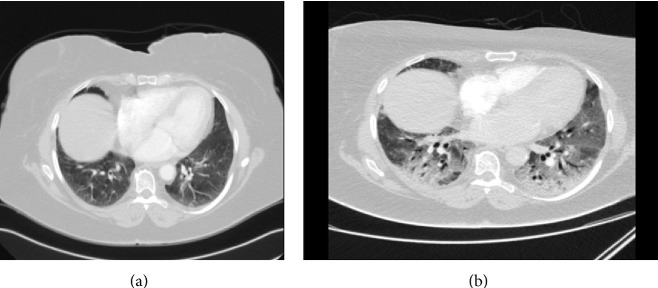
(a) Chest CT scan of patient 1, three months prior to symptoms of pneumonitis. (b) Chest CT scan of patient 1, at the beginning of respiratory symptoms: diffuse interstitial infiltrates and ground glass opacities shown in both lung fields.

A 65-year-old female, never smoker, with a history of hypertension, was diagnosed with colon cancer metastatic to the liver in June 2007. The patient commenced chemotherapy treatment according to the FOLFIRI protocol (bevacizumab/5-fluorouracil/irinotecan). From July 2007 to July 2009, she received 19 cycles of therapy with good response. Then she underwent liver metastasectomy and, in October 2009, she started chemotherapy according to the FOLFOX protocol (bevacizumab/5-fluorouracil/oxaliplatin) [1, 2]. No treatment related side effects were noted and the patient was in very good performance status (WHO 1) until the 6th cycle of chemotherapy. On day 15 of the 6th cycle, she developed dyspnea and fever immediately after the treatment with oxaliplatin. She was treated with intravenous corticosteroids and promethazine with symptomatic improvement of the dyspnea. The following day her dyspnea worsened, with several episodes of near-syncope. Her saturation without oxygen was measured as low as 73%, and blood pressure was as low as 100/50 mm/Hg, but no tachycardia was observed. On physical examination, the patient was dyspneic, rales were heard on auscultation to the lungs, and mild pitting edema was noticed on the lower limbs. Initial blood tests revealed respiratory alkalosis, moderate acute-on-chronic renal failure, and hyponatremia of 122 mEq/L. Troponin and brain natriuretic peptide levels, as well as echocardiography, did not show cardiac failure or ischemia. Chest X-ray showed no pulmonary congestion (following treatment with loop diuretics) or infection. Chest CT scan showed diffused bilateral ground glass infiltrates and no pulmonary emboli (Figure 1). After ruling out cardiac and thromboembolic etiology of the dyspnea, diagnostic bronchoscopy was performed. Bronchoalveolar lavage analysis, along with blood cultures, revealed no sign of bacterial, viral, or* Pneumocystis carinii* infection, and no eosinophilia.* Candida* infection was present in fungal cultures. Pulmonary biopsy showed organizing diffuse alveolar damage. A diagnosis of interstitial pneumonitis was concluded, most probably drug-induced. The patient was treated with high-dose corticosteroids and wide ranging antibiotics, until the diagnosis of bacterial infection was ruled out, antifungal drugs, and loop diuretics. She received respiratory support with oxygen and continuous positive airway pressure. The patient's respiratory failure did not improve with treatment and she died 15 days following initial presentation.

## 3. Case 2

**Figure 2:**
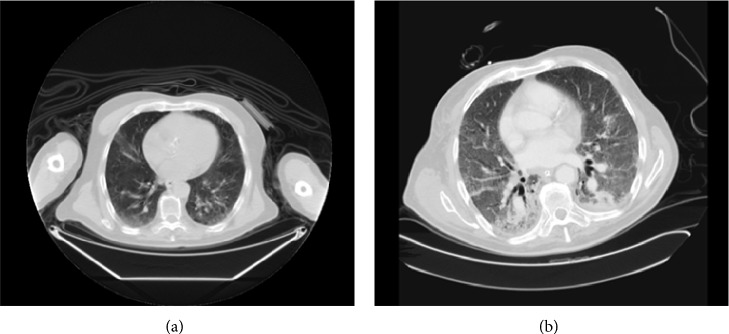
(a) Chest CT scan of patient 2, three months prior to development of respiratory symptoms. (b) Chest CT scan of patient 2, at the beginning of respiratory symptoms: diffuse interstitial infiltrates shown in both lungs.

An 80-year-old male, never smoker, with a history of hypertension was diagnosed in September 2005 with adenocarcinoma of the rectum, stage IIIB. He received standard neoadjuvant treatment of a combination of radiation therapy and 5-fluorouracil and then underwent total mesorectal excision. In July 2006, the patient had local recurrence of the rectal cancer confirmed by rectoscopy, and biopsy from the rectum and inguinal lymph node revealed well-differentiated adenocarcinoma of the rectum, with bilateral inguinal lymph node involvement, a nonresectable disease. CT scan of the chest, abdomen, and pelvis was normal except for the rectal mass and enlarged inguinal lymph nodes. The patient started treatment with chemotherapy for metastatic disease, the FOLFIRI protocol (bevacizumab/5-fluorouracil/irinotecan) for two cycles. Due to the severe side effects of diarrhea, the treatment was changed to FOLFOX protocol (bevacizumab/5-fluorouracil/oxaliplatin). The patient was treated with this protocol for nine months with no side effects reported. Following the 17th treatment cycle, he was hospitalized due to fever, cough, and dyspnea. Physical examination revealed mild dyspnea, and blood tests showed mild respiratory acidosis and acute-on-chronic renal failure. Chest X-ray showed diffuse bilateral interstitial infiltrates. CT scan showed a picture consistent with diffuse alveolar damage without pulmonary fibrosis, with a differential diagnosis of atypical infection or drug-induced pneumonitis (Figure 2). These findings did not appear on the previous CT scan taken three months earlier. Blood and urine cultures were negative. Bronchoscopy including bronchoalveolar lavage was performed. Bronchoalveolar lavage analysis as well as blood cultures revealed no sign of bacterial, viral, or* Pneumocystis carinii*,* Legionella*, and* Aspergillus* infection and no eosinophilia.* Candida* infection was present on lavage analysis. There was no sign of malignancy on bronchial cytology. The patient was treated with wide spectrum empiric antibiotics, glucocorticosteroids, and oxygen, with continuous clinical deterioration and respiratory failure. He died 27 days following his initial presentation.

## 4. Discussion

Oxaliplatin (third generation platin, like trans-L-1,2 diaminocyclohexane, Eloxatin) was first introduced in the year 2000 as part of the treatment in metastatic colorectal carcinoma and demonstrated efficacy both in the metastatic and adjuvant settings [1, 2]. Oxaliplatin demonstrated efficacy in other gastrointestinal malignancies as well, such as gastric and pancreatic cancer. Most common side effects reported in Phase 3 randomized controlled trials were peripheral sensory neuropathy, hematological toxicity, and allergic reactions, including acute laryngeal spasm, mostly at the beginning of therapy, gastrointestinal toxicity, increase in transaminase and alkaline phosphatase levels, and fatigue [3]. Pulmonary fibrosis and grade IV pulmonary toxicity were reported in less than 1% of patients treated in trials that included oxaliplatin, and one patient died of eosinophilic pneumonia [3]. The oxaliplatin prescribing information indicates discontinuation of the drug in any case of unexplained respiratory symptoms, such as nonproductive cough, dyspnea, crackles, or radiological pulmonary infiltrates, until further pulmonary investigation excludes interstitial lung disease or pulmonary fibrosis. Data collected in Phase 4 trials revealed more cases with pulmonary interstitial lung disease. Twenty-six cases of oxaliplatin-related pulmonary toxicity have been described in the English literature [4–21] and are presented in Table 1. Sixteen of these cases (61.5%) were fatal. The real incidence is probably higher and, very likely, only selected cases were described in the literature. Most patients were males (20/26, 77%), were older than 60 years (24/26, 92.3%), with a diagnosis of metastatic colorectal carcinoma (16/26, 61.5%), and were treated with oxaliplatin for less than six months (20/26, 76%). As shown in Table 1, seven of these 26 (27%) patients had previous lung disease, two (8%) were smokers, and 4 (15%) were hypertensive. In the current report, both our patients were hypertensive but none had previous lung disease or smoking history. It was suggested that previous lung disease can be a risk factor for oxaliplatin-induced pulmonary toxicity [15]. However, due to the small number of reported patients, it is difficult to draw firm conclusions regarding such correlation. Of the 26 patients described above, 10 received oxaliplatin as part of adjuvant therapy, and seven died as a result of pulmonary toxicity.

Drug-induced pneumonitis is a diagnosis of exclusion. All the patients described above, including the two patients in this report, underwent extensive workup to exclude the more common causes for pneumonitis: infections, pulmonary emboli, pulmonary bleeding, lymphangitic carcinomatosis, and heart failure. All underwent CT scan and most, including the first patient in this report, underwent bronchoscopy with a lung biopsy that confirmed the less common diagnosis of drug-induced interstitial pneumonitis. Fifteen of the 26 patients were treated according to the FOLFOX protocol (oxaliplatin/5-fluorouracil/leucovorin), and five of the 26 patients were treated with the FOLFOX protocol with the addition of bevacizumab, a vascular endothelial growth factor inhibitor monoclonal antibody. Few incidents of acute lung fibrosis have been reported in patients treated with 5-FU and cisplatin, although 5-fluorouracil is a widely used agent. In two cases of interstitial lung disease that improved with therapy, 5-fluorouracil was reintroduced without additional pulmonary toxicity. This implies that the most likely agent to cause the pulmonary toxicity is oxaliplatin [5, 14].

The mechanism for this pulmonary injury is not yet determined. One study that examined liver specimens of patients with colorectal carcinoma who underwent neoadjuvant chemotherapy and metastasectomy of liver lesions suggested that oxaliplatin may cause sinusoidal injury complicated by fibrosis and veno-occlusive lesions [22]. This injury may be related to oxidative damage and glutathione depletion caused by oxaliplatin. It is possible that this kind of damage may be the pathological base of the pulmonary injury of oxaliplatin.

Some chemotherapy agents are known to cause pulmonary toxicity (bleomycin, busulfan) and have well-established guidelines for follow-up and treatment of this side effect of the drug [23]. There are few case reports on the pulmonary toxicity of oxaliplatin, but this side effect is less recognized. In addition, oxaliplatin is usually given in a multidrug regimen, usually 5-fluorouracil, leucovorin, bevacizumab, or cetuximab; thus the offending agent is not clear. Since this drug is widely administered as the treatment for metastatic colorectal cancer, as well as adjuvant therapy for stage 3 resectable disease and other malignancies, it is important to be aware of this rare but potentially fatal side effect of the drug. It is important to take action for early detection and treatment of this complication.

Our recommendations to reduce the risk of death from pulmonary toxicity of oxaliplatin are as follows:

1. awareness of the potential of pulmonary toxicity of oxaliplatin, which is probably underestimated;
2. discontinuation of the drug in any case of respiratory symptoms associated with radiology findings consistent with interstitial lung injury and considering early treatment with corticosteroids.

In summary, treatment with oxaliplatin for early or metastatic colorectal carcinoma can cause pulmonary toxicity, often fatal, as a rare side effect of the drug. Of the 26 cases reported in the English literature, most patients were males, were older than 60 years, had metastatic disease, and had no previous lung disease. Sixteen patients died of pulmonary toxicity related to oxaliplatin, 10 in the course of adjuvant therapy for resected colon cancer.

## Figures and Tables

**Table 1:** Reported cases of pulmonary toxicity related to oxaliplatin^∗^.

References number	Patient age/gender	Aim of treatment^∗^	Cumulative dose of oxaliplatin (mg/m^2^)	Number of cycles	Regimen^∗∗^	Previous lung disease	Outcome
[4]	60/M	Metastatic disease	910	7	FOLFOX	None	Resolved

[5]	60/F	Metastatic disease	NA	NA	FOLFOX	None	Resolved

[6]	68/F	Adjuvant	510	6	FOLFOX/single agent oxaliplatin	None	Death

[7]	64/M	Metastatic disease (gastric ca)	200	2	FOLFOX	None	Resolved
	75/M	Metastatic disease (gastric ca)	100	1	FOLFOX	None	Resolved

[8]	74/M	Adjuvant	510	6	FOLFOX	None	Death

[9]	67/M	Metastatic disease (HCC)	1100	11	FOLFOX	Pulmonary artery stenosis, lung metastases	Resolved

[10]	62/M	Adjuvant	595	7	FOLFOX	None	Death
	77/M	Metastatic disease	595	7	FOLFOX	None	Resolved

[11]	30/F	Adjuvant	510	6	FOLFOX	None	Resolved

[12]	66/M	Metastatic disease	1020	12	FOLFOX	None	Death

[13]	73/F	Metastatic disease	340	4	FOLFOX	Lung metastases	Death
	71/M	Adjuvant	340	4	FOLFOX	Wegener's granulomatosis, mild COPD	Death

[14]	82/M	Metastatic disease	850	10	FOLFOX	None	Resolved

[15]	71/M	Adjuvant	510	6	FOLFOX	Mild interstitial lung disease	Death
	77/F	Adjuvant	1020	12	FOLFOX	Asymptomatic ground glass opacities at right lung base	Resolved
	69/M	Adjuvant	NA	6	FOLFOX	Asymptomatic subpleural infiltrate	Resolved partially

[16]	76/M	Metastatic disease	260	2	XELOX	None	Death

[17]	47/M	Metastatic disease	NA	NA	XELOX + bevacizumab	Lung metastases	Resolved

[18]	55/M	Metastatic disease	1105	13	FOLFOX	None	Death
	73/M	Adjuvant	765	9	FOLFOX	Emphysematous lungs	Death

[19]	69/F	Metastatic disease	595	7	FOLFOX + cetuximab	Malignant pleural effusion	Death

[20]	73/F	Metastatic disease	1785	11	FOLFOX + bevacizumab	Smoking, suspected lung metastases	Death
	75/M	Metastatic disease (gastric ca)	765	9	FOLFOX	Lung metastases	Death
	64/M	Adjuvant	1020	12	FOLFOX	Smoking	Death

[21]	57/M	Metastatic disease	765	9	NA	None	Resolved

Current paper	65/F	Metastatic disease	1020	12	FOLFOX + bevacizumab	None	Death
	80/M	Advanced locoregional disease	1445	17	FOLFOX + bevacizumab	None	Death

*Note*. ^∗^If not stated otherwise, the patient was treated for colon cancer.

^∗∗^FOLFOX-oxaliplatin/5-fluorouracil/leucovorin, XELOX-oxaliplatin/capecitabine.

ca: cancer.
